# From talking cure to play- and group-therapy: outpatient mental health care for children in the Netherlands *c*. 1945–70

**DOI:** 10.1177/0957154X211024919

**Published:** 2021-07-05

**Authors:** Nelleke Bakker

**Affiliations:** University of Groningen, The Netherlands

**Keywords:** Child Guidance Clinic, children, psychiatry, psychology, diagnostics, Freudianism, group-therapy, Netherlands, outpatient services, play-therapy, psychotherapy, 20th century

## Abstract

After World War II in the Netherlands, outpatient mental health care for children expanded greatly. The number of Child Guidance Clinics grew, and university child-psychiatric clinics and Youth Psychiatric Services were newly established. The leading diagnostic and treatment ideology was mainly Freudian and focused on psychotherapy. During the 1960s the Child Guidance Clinics were outstripped by the more innovative university clinics that introduced new kinds of treatment, such as play- and group-therapy. This ended the hegemony of psychiatrists, as child psychologists and psychiatric social workers replaced them as therapists. At the same time, psychologists of the two denominational university Paedological Institutes took the lead in the scientific study of children’s more serious psychopathology and the development of play-therapy and remedial teaching methods.

## Introduction

From an international perspective, the 1950s and 1960s stand out as the years in which extramural mental health care expanded enormously across the Western world. This development was enabled by the introduction of psycho-pharmacological drugs and was stimulated by the growing criticism of institutional psychiatry, culminating in anti-psychiatry and deinstitutionalization (Shorter, 1997: 239–87). Thanks to the rapid growth of national wealth in that period, the development was stimulated by an increasing willingness of governments to pay for mental health care services. The expansion of outpatient psychiatric services meant that their influence increased from care for mental illness to also include prevention and a wide variety of psycho-social problems. These included street-wandering and alcoholism, which used to be taken care of by welfare provisions of all kinds, organized by the state, churches or other private bodies. In this expanded outpatient sector, in addition to psychiatrists, other professional groups also claimed responsibility, such as clinical psychologists and psychiatric social workers and nurses. These new professions introduced new approaches, usually developed in the USA, such as social casework and, from the late 1960s, behaviouristic psychotherapy and family- and group-therapy, which received minimal attention in the academic training of psychiatrists and consequently reinforced the position of psychologists and social workers (Oosterhuis, 2005a: 256–61).

Little is known as to whether and how these developments were reflected in outpatient mental health care for children in those years. Do we see the same expansion of provisions for children and their parents? Were governments as willing to pay for ‘difficult’ children as they were for adult troublemakers? Were child-rearing problems psychopathologized to the same extent as adult psycho-social problems? Also, were new professions pouring into child-psychiatric services in the same way as they did in adult care? As far as the Anglophone world is concerned, we know that outpatient psychiatric care for children first developed in the late 1920s. It was based on the American model of the Child Guidance Clinic that grew out of the mental hygiene movement, copying both the emphasis on prevention and the belief in the environment as the prime source of mental ill-health (Cohen, 1999: 157–202; Richardson, 1989; Rose, 1985: 197–219; Thomson, 1995, 2006: 109–39; Wright, 2012). This type of clinic was staffed with a multidisciplinary team of a psychiatrist, a psychologist and a psychiatric social worker, and it was closely linked to school health services. The diagnostic and treatment ideology was taken from psychodynamic theory in general. From the mid-1930s and into the post-war era of rapid growth of child guidance, however, psychoanalysis became the predominant theoretical influence (Gleason, 1999; Jones, 1999; Stewart, 2013).

Did these developments also include non-English-speaking countries? As yet, few studies are available in the English language about developments elsewhere. For the Netherlands, it has been demonstrated that the American model of the Child Guidance Clinic was copied there also in the inter-war years, though without psychologists in the multidisciplinary teams and with paediatricians who were charged with the physical examination of a child before (s)he was diagnosed. This explains why the diagnostic and treatment ideology of the Dutch clinics was strongly imprinted not only with dynamic psychology but also with continental physiological character classifications, such as Ernst Kretschmer’s. Another deviation from the American model, inspired by the key role of religion in the Dutch school system, consisted in a lack of interconnectedness of child mental health care with school social work, school health services and school psychologists. Unlike the USA and Scotland, in the Netherlands these school-related services referred to but hardly cooperated with the clinics (Bakker, 2020). Did the Dutch continue on this similar but slightly adapted road into post-war decades?

Another example concerns the development of the Swedish Child and Youth Psychological (from 1960, Psychiatric) Service. This Service was established in 1945 as a result of the developing social-democratic welfare state in which the child symbolized the future, and child care was provided freely as a responsibility of the state, in the way that the British National Health Service was introduced at about the same time (Hendrick, 2003). The Swedish Service expanded rapidly, while focusing on children’s problems defined in terms of ‘social maladjustment’ and addressing the adults surrounding the child. Only from the late 1960s was this social perspective exchanged for a more individualized one that took inspiration from psychodynamic theory, pushing aside biomedical psychiatry and behaviouristic treatments. By 1980, when individual psychotherapy was finally used more often in the treatment of ‘mentally ill’ children, this occurred at a time when the Anglophone world had already moved towards group- and family-therapies and became susceptible to a new neuropsychiatry that linked children’s cognitive and socio-emotional disturbances to the development of their brains (Evans, 2017: 108–41; Zetterqvist Nelson and Sandin, 2013).

After World War II ‘mental health’ replaced ‘mental hygiene’, underscoring that it was important not only to prevent and treat mental problems but also to ensure maximum health and well-being. In 1948 in London, to promote this goal, a World Federation for Mental Health was founded at the International Congress on Mental Health (De Goei, 1998: 67; Oosterhuis, 2005b: 80). Therefore, we may assume that in post-war decades outpatient child psychiatry expanded throughout the West in the way that adult care did. Moreover, it is likely that the 1950s and 1960s saw a general increase in the variety of treatments available to children and parents, in which not only psychiatrists but also child psychologists and psychiatric social workers were increasingly involved. This article addresses post-war developments in child mental health care in the Netherlands, a country that had to choose whether to follow an approach and ideology similar to those in the English-speaking world. Firstly, the organization and development of outpatient child-psychiatric services and the level of responsibility taken by the state will be discussed. These services and their place within a wider network of child welfare provisions – such as school social work, school health services and school psychologists – will be considered, as well as the role of the various professionals involved. Secondly, the article discusses the diagnostic and treatment ideologies that determined the intake procedures and therapeutic practices of these services, as well as their scientific underpinnings.

## Outpatient mental health care for children

In post-World War II years in the Netherlands, outpatient mental health care for children expanded rapidly. At the time, belief in prevention of more serious mental ill-health in adulthood by treatment of children’s behavioural problems reached a peak, and mental health in general was a key interest of politicians and social scientists alike. Next to the material reconstruction of the war-damaged environment, a ‘mental reconstruction’ of the supposedly morally deranged population was believed to be urgently needed. This moral panic was fed by a short period of an unprecedented level of illegitimacy and marriage breakup in the immediate post-war years (Engelen, 1997) followed by two decades of high numbers of children being placed under state guardianship and in homes for delinquent or neglected youth (Dekker, 2012, I: 40–52). Apart from a huge housing shortage (Schuyt and Taverne, 2000: 198–204), juvenile delinquency appeared as the most pressing social problem, and social scientists published worrying reports in the early 1950s on social disruption and wayward youth (Langeveld, 1952; Perquin, 1953) as serious threats to a liberal, Christian and highly divided society. The dividing lines ran first of all vertically, that is between the so-called ‘pillars’ of the large Roman Catholic minority (40 per cent of the population in 1960), the small but influential orthodox Calvinist minority (9 per cent) and the other half of the population consisting of liberal Protestants (28 per cent), other believers (5 per cent) and non-believers (18 per cent). This situation continued until the late 1960s, when secularization and de-pillarization began to increase rapidly (Bakker, Noordman and Rietveld-Van Wingerden, 2010: 275–89).

Despite the widespread moral panic during the post-war years, expectations as to the value of healthy family life rose, while awareness of the possible failure of some parents to live up to professional standards was raised. In particular, the ‘problem’ family – either the ‘antisocial’ or the ‘neurotic’ family – stood in the spotlight of the new social expertise that had developed in the inter-war years. This was focused on preventive interventions by social workers, aimed at keeping parents and ‘maladjusted’ children together instead of institutionalizing the latter. Mental health was a key concern within this ‘therapeutic familialism’ (Rose, 1990: 157–77), in which the Child Guidance Clinics were the only institutions that could boast of a history of practising prevention and focusing on both children and parents. That is why these clinics, rather than other provisions, became the flagship of the Dutch mental health movement. For the Netherlands, the key role of childhood in the revived post-war mental health movement is illustrated by the substantial representation of child psychiatrists at the first National Congress on Mental Health (NCGV) in 1947. At that meeting, four out of seven sections discussed matters of childrearing and education and, unlike other provisions, Child Guidance Clinics were presented as part of the solution for problems threatening social stability (NCGV, 1947).

### Child Guidance and university clinics

The model of the Dutch Child Guidance Clinic was imported from the USA in the late 1920s by Eugenia Lekkerkerker, a young woman jurist who set out to study the treatment of women prisoners there, but came back as a propagandist of child guidance as a prophylactic instrument to prevent young offenders from sliding down towards serious delinquency (Bakker, 2020). The first clinic in the Netherlands was established in Amsterdam in 1928. At first it was called Bureau for Difficult Children, but soon it was felt that this name could deter patients. To avoid the suggestion that the clinic would be an exclusively psychiatric affair, it was decided to call it the *Medisch-Opvoedkundig Bureau* (Medico-Pedagogical Bureau), after clinics that had been established in Berlin and Geneva. The staff consisted of a multidisciplinary team led by a psychiatrist, Nelly Tibout, who had trained in the USA and in Vienna with Rockefeller Foundation grants. The other members were a paediatrician and a psychiatric social worker (Lekkerkerker, 1939). At that time, psychologists were not yet available, and they were not appointed at the Dutch Child Guidance Clinics until after World War II, when they gradually took over the testing. As in the USA, the psychiatric social worker was the only full-time professional in the team – a profession that remained exclusively female until the 1970s.

The seven Child Guidance Clinics that were established before the war employed 15 psychiatrists, 7 paediatricians and 10 licensed psychiatric social workers in 1939 and treated some 3500 children. Another four social workers were training on the two-year course which the Amsterdam School of Social Work offered from 1938; they practised at the two oldest clinics and were tutored by women who had trained in America with a Rockefeller Foundation grant (Lekkerkerker, 1939). Two more social workers were at the time training at the London School of Economics. It is interesting to note that, with two exceptions, the whole first generation of post-war academic child psychiatrists had trained at one of these older clinics (De Goei, 1992: 57–62).

The most rapid expansion of child guidance occurred in the immediate post-war years, when the number of non-denominational clinics grew from 8 in 1946 to 15 in 1952, with consulting hours in three more towns (Van der Grinten, 1987a: 186–209). From 1948, the Roman Catholic Bureaus for Difficult Children were renamed Child Guidance Clinics. The clinics’ formula was also copied, except that – until the mid-1950s – psychiatrists were generally replaced with psychologists due to lack of Roman Catholic psychiatrists (NFGV, 1958: 63–70). In 1960 there were 34 non-denominational, 30 Roman Catholic, and 4 Protestant fully staffed clinics, and 1 Jewish clinic, making a total of 69 clinics; in addition, there were 25 outposts where a travelling team offered consultations less than once a week (NFGV, 1961: 28). By this time, there was full coverage of the country, with all major towns having at least one clinic. This confirmed the status of the clinic and its teamwork as the predominant model of child-psychiatric care. During the 1960s, the network grew at a slower rate; some outposts were upgraded to fully staffed clinics, making up a total of some 80 clinics in the late 1960s (KNBGG, 1967: 25; Van der Grinten, 1987a: 209).^1^ Despite this rapid growth in the number of clinics, waiting lists soon became the rule, a development that confirms the idea that in mental health care the supply creates the demand. Shortages of child psychiatrists and psychiatric social workers continued, thus limiting the proliferation of the clinics. Both types of professionals were trained and tutored at the older clinics.

The first Child Guidance Clinics were financed by private funds only. From 1933, the City of Amsterdam provided additional support to the local clinic, but from 1935 the state undertook much of the financing of the clinics, using the Prevention Fund (*Prophylaxefonds*) for mental health which had recently been established by the Ministry of Health. Next to the state, provinces and cities provided additional funding in varying amounts. In this way, child guidance became part of the public welfare system (Lekkerkerker, 1939). The clinics even succeeded in having the larger part of the state subsidies. In 1950, they obtained 73 per cent of the total amount spent by the Ministry on mental health, but this share diminished subsequently as the adult outpatient sector expanded. Of all state subsidies for mental health care for children in 1950, no less than 90 per cent was spent on the clinics, over one-third of which benefitted the Roman Catholic clinics (Van der Grinten, 1987a: 201). Individual Child Guidance Clinics could add to their financial resources by testing and diagnosing delinquent, neglected and low-achieving children for an advisory report to a juvenile court or an admission committee of a special education school, billing these institutions for this service (Bakker, 2006).

Despite the fact that the Child Guidance Clinics were largely, directly or indirectly, paid from the public purse, they also charged parents small amounts of money, according to their means (Van der Grinten, 1987b: 71–4, 91–100).^2^ They did so because they believed that this would motivate the parents to cooperate with the clinic staff. The clinics’ charging of parents is noteworthy, as all other provisions were free for families and were paid either directly from the public purse or, in the case of privately organized care, subsidized by the Ministries of Health, Education or Justice. This fits the clinics’ profile of a provision meant for the ‘better parents’ – as expressed by Lekkerkerker, the secretary of the National Federation for Mental Health. For her, the clinics served those parents who were ‘caring enough to notice their children’s problems . . . and intelligent enough to understand the meaning of the clinic’ (Lekkerkerker, 1952a: 29–30). This came down to mainly skilled workers and the middle class (Elte, Van Heusden and Frijling-Schreuder, 1953; Lekkerkerker, 1952a; Tibout, 1948: 108; Zwitserlood, 1967).

In the 1930s each of the denominational universities, the Calvinist Free University in Amsterdam and the Roman Catholic University in Nijmegen, had established a Paedological Institute for the diagnosis and treatment of all kinds of ‘difficult’ children. The more serious cases were hospitalized for observation, and the other clients and their parents were seen during consulting hours, usually by a psychologist (Chorus, 2019: 54–85; Rietveld-Van Wingerden, 2006: 45–118; Van Drenth, 2018). The approach of these institutes was similar to that of the children’s departments of the four non-denominational universities’ psychiatric hospitals, established in the 1950s and headed by the newly appointed professors of child psychiatry. These could also either hospitalize a child for observation and treatment or provide outpatient care (Bakker and Smit, 2020; De Goei, 1992: 79–114). Unlike Child Guidance Clinics, these university clinics and Paedological Institutes focused on academic research and development of new kinds of treatment, and both focused on the child itself and his/her problems. An important difference between the two kinds of university provisions, however, is that the child-psychiatric clinics were led by psychiatrists, whereas the Paedological Institutes had a mixed staff in which psychologists predominated and took charge of the research. In the post-war years, academic child psychiatrists and clinical child psychologists trained and learned to do research at these academic centres, whereas the Child Guidance Clinics continued to be the only places where psychiatric social workers learned their profession.

### Referral services

The child guidance and university clinics, as well as the Paedological Institutes, acted as main centres for the diagnosis and treatment of mentally disturbed children. School and toddler health bureau doctors, whose hygienic services likewise came to cover the whole country around 1960 (De Beer, 2008: 69–93; Van Lieburg, 2001: 22–3),^3^ were supposed to refer children with mental problems to one of these clinics or institutes. In the 1950s, school doctors – who were public officials and, up to the 1960s, a largely male profession – refused to take a more substantial role in preventive mental health care, arguing that their assignment of monitoring school children’s physical health was already overloaded and that they were not specialists in that field. This reserve was also due to their fear of entering a heavily contested field that was claimed as religiously sensitive by denominational groups (De Beer, 2008: 141–6).

In contrast, the privately organized, and consequently religiously diverse, and female profession of toddler health bureau doctors opened up their bureaus for special consulting hours for mothers and infants (aged 1.5–6 years) with child-rearing problems, who could see a psychiatric social worker from a nearby Child Guidance Clinic. The screening was done by the social workers, who joined the doctors at their regular consulting hours; this approach was said to prevent maternal resistance, as monitoring a child’s mental development at the same time as his/her physical development was usually welcomed by mothers. This new variety of child guidance outpost was particularly stimulated by the widely supported idea that prevention of mental ill-health should start as early as possible, in toddlers. This was a crucial developmental phase to recognize early symptoms – such as stubbornness, eating and sleeping problems and *enuresis nocturna* – and to intervene through a series of three or four advisory talks with the mother to inform her about ‘normal development’ at this age and help her to adapt her ‘attitude’ to the child. From one experiment in 1950, this practice expanded. In 1960 there were 30 bureaus providing this service (Elte et al., 1953; NFGV, 1961: 25; Noome and Van Wielink-Klimp, 1951).

Shortly after the war, some provincial and city-based social psychiatric services, rooted in adult psychiatric aftercare, opened up a youth department. These Youth Psychiatric Services, seven of which existed in 1961, focused more particularly on the testing of ‘feebleminded’ children and diagnosing more serious behavioural disorders of maladjusted children who might qualify for a special education school or an institution. Their inquiries focused on the child itself and were less extensive than those made by a child guidance team; nor did these services provide treatment. In addition to a psychiatrist, they were staffed with a nurse assistant and a tutor with experience as a teacher at a school for learning-disabled children; from *c.*1960 there was usually also a psychologist for testing. If necessary, the nurse paid a visit to the parents, whereas the tutor advised teachers about children with learning or behavioural problems. Usually the psychiatrist also acted as a member of the admission committee of the local special education school for children who were labelled ‘very difficult to raise’. Most of the clients of the youth departments came, like those of the social psychiatric services in general, from the ‘socially deprived classes’ (Kraft, 1962: 140).

Consultation Bureaus for Child-Rearing Difficulties, which had sprung up from the 1930s, tended to disappear as soon as a Child Guidance Clinic was established in a particular region. This was, for example, the case in the Province of Groningen, where privately organized social health services (*Het Groene Kruis*) had run four such bureaus in the 1950s, staffed by a paediatrician and a nurse, until the province’s capital was provided with a Child Guidance Clinic in 1960. These bureaus had been extensions of the toddler health bureaus, but diagnosed and treated children aged 2–15 years with behavioural problems, particularly *enuresis nocturna* and aggression (Bakker, 2016: 159).

School psychologists were first appointed in the mid-1950s, later than in other Western countries, but their profession developed relatively quickly. In 1956 only two cities had established a school psychological service, but by 1963 no less than 44 services existed, of which 18 were run by Roman Catholic social hygienic organizations. The services focused on ‘children having and causing problems at school’, and they worked closely with school doctors. Like the latter, school psychologists often sat on admission committees for special education schools which – following their appointment – gradually transformed into scientific guidance teams. As a rule, they took charge of the testing of candidates for these schools as part of the selection procedure. At both regular and special schools they supported children with learning difficulties, especially reading problems, developed and tested remedial teaching methods, and supported the work of the newly appointed remedial teachers. School psychologists were often engaged part time by the local Child Guidance Clinic as well. In this way, timely referrals to the clinic of ‘neurotic’ cases – those whose behavioural problems were not caused by learning problems – were guaranteed (NIPP, 1964).

School social work was first introduced in 1946 in Amsterdam, following the American example of visiting teachers. It grew out of a private initiative that sprang from various other services, such as social services, school medical services, school psychological services or Youth Psychiatric Services, a background that created too many different entanglements that thwarted the proliferation of this work.^4^ Like the Youth Psychiatric Services, school social work focused primarily on children and families of the lower social classes, some of which had a history of legal child protection measures. Apart from the social domain, the school social workers were not expected to act on their own accord and they lacked the high status of the psychiatric social workers of the Child Guidance Clinics (Kraft, 1962: 121–2; NVMW, 1957). Like the nurses working at toddler health bureaus, they were, instead, targeted as candidates for a one-year course in child psychiatry to learn when to refer a child to a child-psychiatric clinic or a Child Guidance Clinic. Besides the wide variety of its origins, school social work was impeded more particularly by the key role of religion in the pillarized society; this prevented the development of a common outlook as to the profession’s key aims and of guidelines promoting a common approach to clients, such as those of the National Federation for Mental Health, from which the psychiatric social workers of the Child Guidance Clinics profited so much in terms of a guaranteed high standard for admission to the profession (Oosterhuis and Gijswijt-Hofstra, 2008, I: 686).

## Diagnostic and treatment ideologies

### Diagnosis and its theoretical underpinnings

In the post-war years, with the help of American philanthropy, psychiatry and child psychiatry became more oriented towards psychoanalysis, a development in which Child Guidance Clinics played a key role (Kraft, 1962: 8; Richardson, 1989; Shorter, 1997: 170–89). The first generation of Dutch university professors and lecturers in child psychiatry, who were appointed in the 1950s and led the university clinics, were psychoanalysts who had trained at one of the first Child Guidance Clinics (Bolt and De Goei, 2008: 23). The worldwide approval of the World Health Organization (WHO) report on *Maternal Care and Mental Health* by the British child psychiatrist John Bowlby (1951/1952) reinforced the focus of the mental health movement on early childhood as a risky phase and on warm and continuous maternal care during infancy as a crucial condition to prevent mental ill-health. The psychoanalytic movement, which had moved from Vienna to London and the USA, likewise reoriented towards the mother–infant relationship (Zaretsky, 2004: 249–75), a shift that fitted the conservative Netherlands of the 1950s with its predominantly stay-at-home mother perfectly (Bakker, Noordman and Rietveld-Van Wingerden, 2010: 282–5). Bowlby’s ideas about the pathogenicity of ‘maternal deprivation’ became so popular that they were used to criticize the widely practised welfare policy of sending poor school-children and toddlers to health colonies for six weeks, during which hardly any contact with their parents was allowed (Bakker, 2007). Then in 1953, after a flood had inundated large parts of the coastal province of Zeeland, the government asked for advice from the president of the National Federation for Mental Health about proper procedures for the possible evacuation of the population. He advised against separation of parents and young children (Oosterhuis and Gijswijt-Hofstra, 2008 I: 651).

In addition to psychodynamic theory and a focus on the environment as an explanation of a child’s mental ill-health, multidisciplinary teamwork and a strong emphasis on prevention were the essence of the Child Guidance Clinic’s approach. As in the pioneering English-speaking countries, the link with delinquency prevention soon weakened and was replaced with a narrower focus on the family itself. The clinics’ staff claimed that treatment of a problem child and his/her parents implied prevention of problems of the other children in the family: healthy parents had healthy families, and the proper care of children was, by nature, prevention (Frijling-Schreuder and Petri, 1962; Hustinx and Snellen, 1959).

The importance of teamwork is shown especially in the process of diagnosis. At the Child Guidance Clinics, this was complicated and time-consuming. A referred child was first examined by the paediatrician, who looked for constitutional abnormalities; next, the child was tested by the psychiatrist or psychologist, who diagnosed his/her mental capacities compared with normal development. The testing concerned intelligence, school attainment, language, attention, accuracy, motor ability, artistic expression, character, understanding of human situations, and special talents. Intellectually ‘subnormal’ children were excluded from treatment. Meanwhile, the psychiatric social worker prepared the anamnesis by making home visits to gather information from the parents, and also spoke with the teacher and the family’s general practitioner. The psychiatrist and team-leader talked with the child two or three times, observing his/her play and interpreting the test results concerning the child’s abilities, character and problems. The diagnosis of what was troubling the child and advice as to further treatment were prepared in a ‘staff conference’ of all team members who had seen the child. The social worker wrote an advisory report which evaluated the child’s development and prospects, as well as the parents’ suitability for what was called *milieu* treatment. The report was sent to the referring professionals and communicated orally to the parents (Frijling-Schreuder and Van der Velde, 1963; Tibout, 1948: 25–101).

Referrals to Child Guidance Clinics were most often made by (school) doctors, schools, juvenile judges and guardianship societies, with the share of the doctors and schools increasing in the 1950s and 1960s and that of judicial children’s care sharply decreasing. About one-fifth of the children were brought by the parents of their own accord. The characteristics of the referred children remained remarkably stable over time; they were of primary school age (6–11 years) and two-thirds were boys. In asking for help for their children, parents most frequently mentioned as reasons: learning problems, aggressive behaviour, and anxieties or nervousness. These categories remained the most common, with aggression taking over the lead from learning problems in the 1960s (Lubach, 1974: 36–8; MOB Zaanstreek, 1948: 25; Tibout, 1948: 106–7). They were similar to the problems that brought parents and children to American Child Guidance Clinics (‘the everyday problems of the everyday child’; Jones, 1999: 91–5). On average, these learning and behavioural problems were similar but less serious than those for which the outpatient departments of the university child-psychiatric clinics catered (Bakker and Smit, 2020), whereas the Youth Psychiatric Services and the Paedological Institutes took care of the more serious cases of learning disabilities and autism (Chorus, 2019: 54–89; Rietveld-Van Wingerden, 2006: 45–118; Van Drenth, 2018).

Diagnoses differed between institutions. At first, the Child Guidance Clinics interpreted almost every complaint in psychoanalytic terms, as caused by a child’s unconscious inner conflict (neurosis) or neurotic interaction between parent and child, rooted in parenting faults that were often blamed on the mother’s own neuroticism (Frijling-Schreuder, 1953, 1955: 43–64; Tibout, 1948: 201–14). Although ‘affective neglect’ and other ‘family problems’ continued to be the most important causes, the files of the Arnhem clinic show that in the 1960s organic defects such as brain damage were conceived as the most important cause of the trouble in no less than one-quarter of the diagnosed cases (Lubach, 1974: 39).

At the Youth Psychiatric Services, the admission process was much simpler and was usually done by a social psychiatric nurse, who also contacted the referring school. A psychiatrist or psychologist took care of the testing, diagnosis and referral of a learning-disabled or otherwise ‘difficult’ child to a special school or an institution (Kraft, 1962: 142–7). In contrast, the Paedological Institutes tested children extensively and sometimes hospitalized a child for long-term observation. These institutes’ practices were guided by developmental psychological theory of all kinds, which led them to diagnose developmental, neurological and perceptual disorders, such as mental retardation, ‘wordblindness’, restlessness and infantile autism. Their admission procedures were science-based and focused almost exclusively on testing a child’s mental capacities, such as intelligence, perception, attention, motor ability, artistic expression and character (Chorus, 2019: 54–85; Rietveld-Van Wingerden, 2006: 45–118; Van Drenth, 2018). At the non-denominational university clinics, however, diagnosis was an exclusively psychiatric assessment and was done by a single psychiatrist. Although academic child psychiatry was equally dominated by psychodynamic theory, these clinics more often saw organic, particularly neurological, causes as well as neuroticism. This openness to other causes of children’s problems relates to the medical environment where the intake proceeded: a university hospital with opportunities for more sophisticated physiological and neurological examinations, such as an electroencephalogram (Bakker and Smit, 2020).

From the late 1950s, the time-consuming multidisciplinary admission and diagnostic procedure of the Child Guidance Clinics and the way the teams stuck to these principles stimulated criticism among both the public and referring institutions. This was reinforced by the increasingly long waiting lists. Child guidance professionals, however, firmly defended their approach and even downplayed other, more ‘pedagogical’ means of intervention (Frijling-Schreuder, 1965; Lekkerkerker, 1962; Redactie, 1964). It was often repeated that ‘simple cases do not exist’. In defence of their procedure, the extensive information gathering by the psychiatric social worker for an anamnesis was tested and found to be superior by the team of the Delft clinic. An experiment at this clinic demonstrated that parents were more open in talking to the psychiatric social worker than to the psychiatrist, because parents were usually fearful of the latter rather than trusting. This team also experimented with a shorter admission procedure by a single professional and concluded that a ‘partial examination’ should only be done in cases of learning problems (by a psychologist) or physical complaints (by a paediatrician). Some cases had turned out to be more complicated and had therefore been examined by the whole team (Hustinx and Snellen, 1959). Another time-saving measure with which teams experimented in the late 1960s was using the same admission procedure at a Child Guidance Clinic and a Youth Psychiatric Service (Hart de Ruyter, 1965a; Zwitserlood, 1967).

This combined admission procedure was one of the suggestions to reduce waiting lists made by Theo Hart de Ruyter at a conference on the future of the Child Guidance Clinics in 1965. He was the first Dutch full professor of child psychiatry, a psychoanalyst, a former leader of each of the two kinds of institution involved, a highly respected authority in the field (Bakker and Smit, 2020) and himself an early practitioner of this combined method. He also subscribed to the idea that not all cases needed an extensive, ‘classic’ examination; in many cases a shorter, ‘modern’ one could suffice (Hart de Ruyter, 1965a). He warned that the waiting lists were scaring away referring institutions, with the consequence that others had to take care of many more cases, and he told the audience that the psychiatric language of the advisory reports irritated non-medical professionals. He explained that juvenile judges and guardianship societies preferred to have children diagnosed by Youth Psychiatric Services, because they thought the Child Guidance Clinics were ‘unwieldy and slow’. Moreover, he thought that the clinics too often blamed their huge backlog of indicated – but not yet treated – cases on the shortage of personnel and avoided the more difficult cases of children who ‘acted out’. Hart de Ruyter’s critique was sharp and focused particularly on the clinics’ inflexibility, inefficiency and rigidity that made them less efficient than other institutions.^5^ At the same conference, BJG Bremer, a psychologist and the leader of the first Roman Catholic Child Guidance Clinic, warned against the dangerous spirit of ‘splendid isolation’ that he felt among his peers. The clinics should, instead, become centres of expertise and training and should connect with and support other institutions (Bremer, 1965).

### Treatment ideologies

It was a deliberate strategy of the Child Guidance Clinics only to accept for treatment children of normal intelligence, who were on the one hand ‘not too disturbed’ and on the other hand disturbed enough for ‘more simple methods’ to be ineffective (Lekkerkerker, 1952b: 230). As a consequence, over half of the referred children were not treated at the clinics; they made up the ‘advice cases’ (Lekkerkerker, 1952a: 31). This heterogeneous category encompassed: (1) clear-cut cases of a sickening organic condition; (2) ‘social’ cases (related to housing or unemployment) that were referred to social workers; (3) delinquent or neglected children, about whom advisory reports were sent to a juvenile judge or a guardianship society; and (4) children diagnosed as ‘feeble-minded’, ‘very difficult to raise’ or suffering from a ‘partial learning defect’ such as a serious reading problem, all of whom were referred to a special education school. Other, less complicated cases were given a maximum of three talks (a ‘short treatment’) with the mother to help her change her ‘attitude’, for example in cases of ‘neurotic interplay’ between herself and her jealous or anxious toddler (Frijling-Schreuder, 1955: 43–64; Lekkerkerker, 1952a: 17, 31; 1952b: 229–40). The exclusion of ‘social’ cases – in the widest sense – reinforced the profile of the clinics as being for families of skilled workers and the middle class. The required cooperation of the parents and the focus on talking with them probably had the same effect.^6^

Full, long-term and therefore relatively costly psychoanalytic treatment at a Child Guidance Clinic consisted of a talking cure. It was provided to ‘normal’ children who had, because of environmental circumstances, temporarily drifted away from normal development. They were usually diagnosed as victims of a neurosis, no matter what the symptoms were; these could be anything, from learning to eating problems or from anxiety to lying or stealing. The clinics’ psychiatrists emphasized that treatment and diagnostic observations and talks were inextricably linked: the treatment followed the advisory report and it might, in turn, cause a change to the prognosis. Child guidance psychiatrists held that the treatment actually started during the initial phase of diagnostic observation, when the psychiatrist started to build a trustful relationship with the child, and the psychiatric social worker set out to establish one with the parents (Frijling-Schreuder, 1953, 1965; Lekkerkerker 1952b: 293–301).

For psychotherapy, a child came to the clinic twice a week, talking with the psychiatrist and, in the case of a young child, also playing or drawing. The therapist invited the child, in a non-directive way, to reveal his/her unconscious feelings about his/her environment, for example by drawing or telling stories. At the same time the psychiatric social worker practised the casework-based ‘milieu treatment’; this involved more talks with the parents, or just the mother, focusing on the desired change of attitude by taking away unconscious barriers to accepting a child and showing affection, or by limiting overprotection. The social casework method, which was developed in American psychiatric social work and relied on a carefully built relationship of trust, aimed to solve problems by talking about them and encouraging clients to face up to motives behind their behaviour in order to improve self-knowledge and self-reliance (Oosterhuis, 2005b: 82–3). This implies that, as in the pioneering English-speaking countries, treatment largely came down to guidance of parents. Theoretical inspiration for psychotherapy with children was initially taken from the London school of analytic child psychiatry, particularly the work of Anna Freud, whose key concepts – such as ‘infantile affective neglect’, ‘Ego weakness’ and ‘defence mechanisms’– were omnipresent in the child guidance psychiatrists’ publications. Other favourites were Bowlby, Susan Isaacs and the Austrian youth psychiatrist August Aichhorn (Hart de Ruyter, 1956, 1959a; Tibout, 1948: 273–312).^7^

Hart de Ruyter, head of the Groningen University child-psychiatric clinic, was the leading Dutch theorist on psychoanalytic treatment of children in the 1950s and 1960s. He was also the author of an authoritative textbook on child psychology and psychiatry for students in (special) education, social work and psychiatric social nursing that saw seven editions between 1952 and 1968 (Hart de Ruyter, 1952, 1965b). His theoretical work shows a clear therapeutic optimism; he defended the possibility of successful psychiatric treatment of even the most difficult cases. Through therapy, he claimed, he could go back to the pre-oedipal stage (between eight months and three years of age), marked by unfulfilled hunger for affection, and redress the child’s adverse emotional development. He maintained that many of the most difficult cases were ‘affectively neglected’ neurotics, who were ill but curable and needed psychiatric treatment to reinforce their weak Ego. Illness of this kind manifested itself, according to him, especially during the pre-adolescent stage, between 10 and 12 years of age, when a healthy Ego and Super-Ego were bound to mature (Hart de Ruyter, 1951, 1956, 1959a, 1959b).

Whereas the child guidance psychiatrists took their inspiration almost exclusively from psychoanalysis, Hart de Ruyter preferred to use more varied methods from apparently incompatible psychological and psychiatric theory from across the Western world. According to him, inborn physical and mental characteristics, such as a child’s temperament, mattered in that some personality types were more susceptible to mental illness than others (Hart de Ruyter, 1955: 21–9). Regarding the stages of development, however, he showed himself a real Freudian in distinguishing the oral, anal, phallic, oedipal, latent, and (pre-)adolescent stages, during which a child progressed from the first impulses towards gratification to a gradual sublimation of lust based on the love for his/her mother. As a result, the Ego could develop towards adaptation of his/her behaviour to the demands of the wider community, that would be internalized by his/her developing Super-Ego (Hart de Ruyter, 1955: 38–94).

Anna Freud was Hart de Ruyter’s prime source of inspiration concerning the analysis of a child, the coping strategies (‘defence mechanisms’) a child might develop, as well as the determination of a(n) (un)healthy development by infantile affective relationships and the need for Ego-reinforcement (Hart de Ruyter, 1959a). Regarding early childhood, he adopted theories of other psychoanalysts as well, particularly Melanie Klein, Margaret Ribble and René Spitz. They all emphasized – although in different ways – the infant’s essential need for motherly love and affection (Hart de Ruyter, 1952: 32–43, 130–51).^8^ He linked up these ideas with the views of the Viennese psychoanalyst August Aichhorn, who worked with institutionalized youths and diagnosed them as ‘affectively neglected’ during infancy because of a broken home or having been raised in a children’s home. Soon after Bowlby’s WHO report had first appeared in 1951, Hart de Ruyter (1955: 126) referred to the ‘undeniable connection between affective neglect in the first years of life and disorder of the conscience’ as a well-known fact among child psychiatrists that was now statistically verified.

Following Bowlby, Hart de Ruyter (1959a) insisted time and again that most behavioural problems (he estimated that it was three-quarters) had their origin in ‘early affective neglect’, which had frustrated the healthy growth of ‘basic security’ in the pre-oedipal stage of development. The neuroticism that was thus created could be treated successfully with psychotherapy. For a young child, play-observation and drawings of trees and family members were indicated as diagnostic and therapeutic instruments to make a child show his/her unconscious feelings and unresolved conflicts. School children could likewise be ‘opened up’ by drawing their families, interpreting Rorschach inkblots and telling stories, while (pre-)adolescents could tell and write down fantasies about their future, and also complete stories and sentences to find out what unconscious feelings they were struggling with (Hart de Ruyter, 1951, 1956, 1959b). These instruments would guarantee continuity between diagnosis and treatment. As in his theoretical preferences, Hart de Ruyter showed more openness to new therapeutic approaches than his child guidance colleagues did. Like the head of the Utrecht University’s child-psychiatric clinic, Lucas Kamp (1954), he was, for example, an early user of play-therapy for young children, and group-therapy for school-children and adolescents. The latter allowed for an analysis of their emotions and social skills and could promote Ego reinforcement, Hart de Ruyter claimed (1955: 175–8; 1956; see also Mik, 1969). Moreover, in his Groningen clinic he used medication not only in cases of epilepsy but also as a means to support psychotherapy’s effectiveness (Bakker and Smit, 2020).

The Roman Catholic Child Guidance Clinics and their Paedological Institute did not use psychoanalysis, except for Bowlby’s extension of the theory into the crucial role of maternal love and accessibility during infancy (CKMOB, 1956; Trimbos, 1955: 7–21). Non-orthodox varieties of psychoanalysis, especially those from which the sexual dimension was excluded, such as Alfred Adler’s Individual Psychology, were accepted as therapeutically useful (Bakker, 2014). In general, their focus was directed towards treatment by psychologists and educationalists trained at the Nijmegen University’s Paedological Institute, which pioneered not only child (neuro)psychology but also cutting-edge research into constitutional causes of learning and behavioural problems such as autism (Van Drenth, 2018). Compared with the non-denominational Child Guidance Clinics, the Roman Catholic staff were much more open to new approaches in treatment. For example, while consistently declined by mainstream child guidance psychiatrists as too pedagogical, play-therapy was introduced as early as 1948 by Bernard Bremer, the leader of a Roman Catholic Consultation Bureau for Child-Rearing Difficulties, after taking a course in London (Westhoff, 1996: 147). Play-therapy was also developed, both practically and theoretically, at the orthodox Calvinist Free University’s Paedological Institute. Their scientific research was likewise more oriented towards constitutional and neurological causes of learning and speech problems and to science-based methods of testing, remedial teaching and various other kinds of ‘paedotherapy’ (Rietveld-Van Wingerden, 2006).

Although at the Roman Catholic Child Guidance Clinics, psychiatrists were the official leaders of the teams, their psychologists were more influential and led the development of new approaches such as play-therapy and group-therapy for children. These therapies played the same role as the newly introduced Rogerian therapy (‘counselling’), behavioural therapy and *Gestalt-*therapy in adult psychiatry. They took less time, were much cheaper than the psychoanalytic ‘talking cure’ and had the effect of breaking the therapeutic privilege of psychiatrists as they were applied by psychologists. Therefore, it was no coincidence that a Roman Catholic child psychiatrist expressed his fear in 1957: ‘The psychiatrist becomes a superfluous . . . teammate’ (Lumey, 1957: 18).

Among non-denominational child guidance psychiatrists, it took some time to adjust to these developments. Experiments with shorter and cheaper kinds of psychotherapy, such as seeing a child only once a week in cases of trauma or adolescent developmental-phase problems, did not start until around 1960 (Frijling-Schreuder, 1965; Hustinx and Snellen, 1959). Some clinics experimented with new approaches, such as group-therapy for adolescents, creative expression therapy and parent discussion groups, all of which could be applied by a psychologist or a psychiatric social worker (De Bel, 1963; Van Westering-Visser, 1963). However, the final blow to the child guidance psychiatrists’ status did not come until the late 1960s. The introduction of American-style family- or system-therapy across the child mental health sector finally brought together parents and children in a single therapeutic process, with a psychiatric social worker as therapist (Blankstein and Van Veen, 1968). Child psychiatrists had to wait for the arrival of the new biological psychiatry of the 1980s to regain their key position in a reorganized mental health care landscape.

## Conclusion

As in the Anglophone world, from which the Dutch imported their model of Child Guidance Clinics in the late 1920s, in the Netherlands the original link with the prevention of delinquency weakened soon and was replaced with a focus on the family itself. In the post-war years, outpatient mental health care for children expanded rapidly. In addition to Child Guidance Clinics, university child-psychiatric clinics and Youth Psychiatric Services opened. Unlike these services, the Child Guidance Clinics took on a profile of being for families of skilled workers and the middle class, by emphasizing the ‘normality’ of their patients, excluding ‘social’ cases, requiring parental cooperation and providing only talking cures. They focused, moreover, on the less serious cases of mental illness, particularly neurotics. This implied that other institutions had to take on the treatment of more serious cases of learning and behavioural problems. This was done at the university clinics and the two denominational Paedological Institutes. Prophylactic health services, such as toddler health bureaus and school doctors, referred patients to the child guidance and university clinics and to the Paedological Institutes for diagnosis, advisory reporting and treatment. The leading diagnostic and treatment ideology of the non-denominational child guidance and university clinics was rather one-sidedly Freudian, finding inspiration particularly with Anna Freud’s ideas about psychoanalytic diagnostics and treatment of children.

From the 1960s, however, the Child Guidance Clinics were outstripped by more efficient, flexible and innovative institutions, such as the university clinics that used a shorter and simpler admission procedure and a greater variety of treatments, including new therapies. With time, the psychoanalytic talking cures of the Child Guidance Clinics, psychotherapy for the child and *milieu* treatment of the parents were more often replaced with cheaper kinds of therapy, such as play-, group- and family-therapy. As in adult psychiatry, this in turn ended the hegemony of psychiatrists, as they were replaced with child psychologists and especially psychiatric social workers as therapists. However, even before psychoanalysis lost its predominance, the psychologists of the Paedological Institutes of the two denominational universities had taken the lead in the scientific study of children’s more serious psychopathology and the development of innovative, science-based varieties of play-therapy and remedial teaching methods.

## Notes

1. Because sources are incomplete, an exact number cannot be given. In 1966, 34 Roman Catholic clinics existed according to KNBGG (1967: 25). Van der Grinten (1987a: 209) mentions that the number of clinics did not change significantly after 1962, when he counted 75 clinics.

2. In 1946 the parental contribution amounted to no more than 7 per cent of the total costs; Van der Grinten, 1987b: 72.

3. Local communities and regions appointed school doctors. In the 1950s, only some rural areas still did not have this service; in 1965 the network was completed; De Beer, 2008: 69–93. Private societies set up infant and toddler health bureaus. There were *c.* 1,500 in 1950 and 3,000 in 1970; Van Lieburg, 2001: 22–3. In the 1950s, some 70 per cent of Dutch infants were monitored by the doctors of these bureaus; Bakker, Noordman and Rietveld-Van Wingerden, 2010: 310.

4. After 10 years, only 16 services had been established; NVMW, 1957: 8.

5. In 1964 in the City of Groningen, the Youth Psychiatric Service dealt with *c*. 500 cases, the university clinic with *c*. 400 cases, and the Child Guidance Clinic with no more than 100 cases; Hart de Ruyter, 1965a: 289.

6. In Amsterdam in 1946 only 21 per cent of the fathers of the referred children were unskilled workers, 33 per cent were skilled workers, 28 per cent were lower middle-class employees, and 18 per cent upper middle-class employees; Tibout, 1948: 108. In Zaandam in 1947, these percentages were 25, 45, 50 and 6, respectively; MOB Zaanstreek, 1948: 25. In Arnhem in 1963 and 1971, these percentages were on average: 15, 12, 32 and 23, respectively, with another 22 per cent ‘middle-class’ employees; Lubach, 1974: 29.

7. These were also the authors whose main publications on psychoanalytic treatment of children were translated in the 1950s and to whom Dutch authors referred.

8. In his textbook’s chapter on the pre-oedipal phase, he referred to René Spitz and Melanie Klein, and in the chapter on ‘Child-rearing faults’ he referred to Margaret Ribble. For the differences between their theories, see Geissmann and Geissmann, 1998: 77–132.
